# Early development of *Moniliophthora perniciosa *basidiomata and developmentally regulated genes

**DOI:** 10.1186/1471-2180-9-158

**Published:** 2009-08-04

**Authors:** Acássia BL Pires, Karina P Gramacho, Delmira C Silva, Aristóteles Góes-Neto, Mylene M Silva, Jairo S Muniz-Sobrinho, Ricardo F Porto, Cristiano Villela-Dias, Martin Brendel, Júlio CM Cascardo, Gonçalo AG Pereira

**Affiliations:** 1 Centro de Biotecnologia e Genética, Laboratório de Genômica e Expressão Gênica, Departamento de Ciências Biológicas, Universidade Estadual de Santa Cruz, Rodovia Ilhéus-Itabuna, km 16, 45662-000, Ilhéus-Bahia, Brazil; 2 Centro de Pesquisas do Cacau, CEPLAC, Rodovia Ilhéus-Itabuna, km 22, Ilhéus-Bahia, Brazil; 3 Laboratório de Pesquisa em Microbiologia (LAPEM), Universidade Estadual de Feira de Santana, Av. Transnordestina, s/n, bairro Novo Horizonte, 44036-900, Feira de Santana, Bahia, Brazil; 4 Laboratório de Genômica e Expressão, Departamento de Genética e Evolução, Instituto de Biologia, Universidade Estadual de Campinas, CP 6109, Campinas, São Paulo, Brazil

## Abstract

**Background:**

The hemibiotrophic fungus *Moniliophthora perniciosa *is the causal agent of Witches' broom, a disease of *Theobroma cacao*. The pathogen life cycle ends with the production of basidiocarps in dead tissues of the infected host. This structure generates millions of basidiospores that reinfect young tissues of the same or other plants. A deeper understanding of the mechanisms underlying the sexual phase of this fungus may help develop chemical, biological or genetic strategies to control the disease.

**Results:**

Mycelium was morphologically analyzed prior to emergence of basidiomata by stereomicroscopy, light microscopy and scanning electron microscopy. The morphological changes in the mycelium before fructification show a pattern similar to other members of the order *Agaricales*. Changes and appearance of hyphae forming a surface layer by fusion were correlated with primordia emergence. The stages of hyphal nodules, aggregation, initial primordium and differentiated primordium were detected. The morphological analysis also allowed conclusions on morphogenetic aspects. To analyze the genes involved in basidiomata development, the expression of some selected EST genes from a non-normalized cDNA library, representative of the fruiting stage *of M. perniciosa*, was evaluated. A macroarray analysis was performed with 192 selected clones and hybridized with two distinct RNA pools extracted from mycelium in different phases of basidiomata formation. This analysis showed two groups of up and down-regulated genes in primordial phases of mycelia. Hydrophobin coding, glucose transporter, Rho-GEF, Rheb, extensin precursor and cytochrome p450 monooxygenase genes were grouped among the up-regulated. In the down-regulated group relevant genes clustered coding calmodulin, lanosterol 14 alpha demethylase and PIM1. In addition, 12 genes with more detailed expression profiles were analyzed by RT-qPCR. One aegerolysin gene had a peak of expression in mycelium with primordia and a second in basidiomata, confirming their distinctiveness. The number of transcripts of the gene for plerototolysin B increased in reddish-pink mycelium and indicated an activation of the initial basidiomata production even at this culturing stage. Expression of the glucose transporter gene increased in mycelium after the stress, coinciding with a decrease of adenylate cyclase gene transcription. This indicated that nutrient uptake can be an important signal to trigger fruiting in this fungus.

**Conclusion:**

The identification of genes with increased expression in this phase of the life cycle of *M. perniciosa *opens up new possibilities of controlling fungus spread as well as of genetic studies of biological processes that lead to basidiomycete fruiting. This is the first comparative morphologic study of the early development both *in vivo *and *in vitro *of *M. perniciosa *basidiomata and the first description of genes expressed at this stage of the fungal life cycle.

## Background

*Moniliophthora perniciosa *(Stahel) Aime and Phillip-Mora (2005) [1] is a hemibiotrophic basidiomycete that causes Witches' Broom Disease (WBD) in cocoa (*Theobroma cacao *L). Currently, WBD occurs in South and Central America and can cause crop losses of up to 90% [2]. In Bahia (Brazil), *M. perniciosa *was identified in 1989 [3] and, as a consequence of its spreading, the annual production of cocoa beans dropped from 450,000 to 90,000 tons within 12 years, reducing export values from an all-time high of about US$ 1 billion to 110 million. During this period nearly 200,000 rural workers lost their jobs, resulting in an intensive migration from farms to urban areas [4].

The fungus infects young meristematic tissues inducing hypertrophy and hyperplasia, loss of apical dominance, and proliferation of axillary shoots. The hypertrophic growth of the infected vegetative meristems (green broom) is the most characteristic symptom of WBD [5]. Basidiomata, in which basidiospores are produced, develop on dead but attached dry brooms of cacao trees in the field, after dry and wet periods. Basidiospores are spread by wind and depend on sufficient moisture for survival. They can only germinate on and infect susceptible cacao tissues (i.e. buds, young leaves, flower cushions, or young pods) if relative humidity levels are near 100%. Shortly after infection the pathogen establishes a biotrophic relationship with the host during which the fungus has an intercellular, biotrophic, monokaryotic growth phase, without clamp connections. Four to six weeks later, the hyphae become dikaryotic, develop clamp connections and the fungus grows saprophytically [5]. A well-characterized concerted series of cell death events [6] causes the green broom to become necrotic, and basidiomata are formed in a favorable environment after 6 weeks or more [7].

Information about morphological development and environment that affect basidiomata and basidiospore production of *M. perniciosa *are important to improve the *in vitro *culture of the pathogen and to study its life cycle. Environmental conditions for basidiomata production have been described by Suarez [8], Rocha [9] and Rocha and Wheeler [10,11]. An artificial production of basidiomata has been studied by several authors, but an ideal production mode has not yet been achieved. Stahel [12] observed basidiomata development on mycelial mats in agar cultures. Purdy *et al. *[13] and Purdy and Dickstein [14] modified Stahel's methods to produce basidiomata on mycelial mats. Griffith and Hedger [7] improved basidiomata production by using bran-vermiculite medium, a method currently used to produce *M. perniciosa *basidiospores. Later, Niella *et al*. [15] modified medium formulation and Macagnan *et al*. [16] removed vermiculite and the extra layer of cacao powder and CaSO_4 _originally used to cover the medium and to reduce the time to fruiting. The difficulty of obtaining axenic cultures and the long cultivation time has hindered more detailed studies on the morphology and early development of *M. perniciosa *basidiomata.

Several studies of basidiomata development in other basidiomycetes, e.g., *Agaricus bisporus*, *Flammulina velutipes*, *Boletus edulis *[17] as well as mycorrhizal fungi such as *Laccaria *sp. [18] have already been published, complementing research on *Coprinopsis cinerea *and *Schizophyllum commune*, which are models for developmental studies in macroscopic basidiomycota [19]. Basidiomata of *M. perniciosa *produced either in nature [20-22] or under laboratory conditions [13,7,14] have been studied and their morphology was originally described by Stahel [12]. Later, Delgado and Cook [23] showed that the hyphae found in basidiomata are dikaryotic whereas basidia are monokaryotic (i.e. diploid, following karyogamy).

Although the microscopic characteristics and growth patterns of both monokaryotic and dikaryotic mycelia have been described elsewhere [24-26], there is no microscopic characterization of the pattern of basidiomata development. We provide the first description of primordium development of *M. perniciosa *basidiomata. Based on our observations the development was divided in four stages, similar to those described for *A. bisporus *(17). Together with the sequencing and annotation of the *M. perniciosa *genome [27], detailed morphologic information is important for future research into *M. perniciosa *mutants, complementing genetic studies. Here we describe and histologically compare the development of both *in vivo *and *in vitro*-grown *M. perniciosa *basidiomata and analyze the expression of 192 selected ESTs by macroarray and of 12 ESTs by RT-qPCR.

## Results and discussion

### Morphological observations

**Figure 1:**
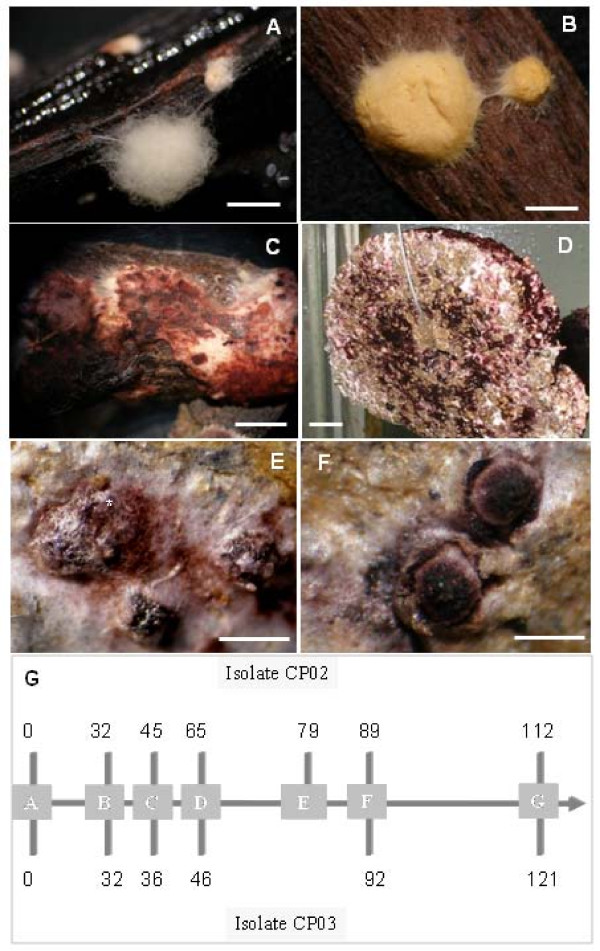
**Mycelial stages prior to emergence of *M. perniciosa *primordia**. A, B, C. Mycelial mat originating from basidiospore germination on dead cocoa branches. D. Mycelial mat cultured on artificial substrate. Mycelium is initially white (A) then turns yellow (B) and changes to reddish pink (C) (A, B, C; bars = 0.5 cm), and maintains this color during primordial and basidiomata development, both in natural and artificial conditions (D; bar = 1.25 cm). E. Globose protuberance covered by mycelial mat (*) and openings for initial sprouting (bar = 1 mm). F. Primordia emergence (bar = 1 mm). G. Schematic representation of the sampling during cultivation for library construction (CP03) and macroarrays and RT-qPCR (CP02). Lateral numbers indicate days of cultivation. Box A – time 0, when the Petri dishes were inoculated. Box B – First harvest before hanging the mycelia in moist growth chambers. Box C – Second harvest with yellow mycelia. Box D – Third harvest with pink-reddish mycelium. Box E – Fourth harvest with reddish-pink mycelium before stress. Box F – Fifth harvest with dark pink mycelia (CP03), or reddish-pink after stress (CP02). G – Sixth harvest of primordia and fully-developed basidiomata. The days of cultivation differ due the differences between fungal isolates.

Observations of dead brooms kept in humid chambers or collected directly from the field showed the presence of a thin mat of saprophytic mycelium on the surface of the brooms. It was possible to notice color changes and the morphology that preceded basidiomata formation on this mat. The aerial mycelium formed a thick layer with notable color modifications: it was initially white (Figure 1A), then yellow (Figure 1B) and later, reddish pink (Figure 1C). At a later stage, dark-brown to reddish spots appeared until onset of primordium growth (Figure 1E and 1F). The same characteristics were observed in artificial cultivation (Figure 1D), which allowed a monitoring of the morphogenetic stages of *M. perniciosa *basidiomata.

Currently two media are used to produce basidiomata of *M. perniciosa*. The "Griffith medium" [7] contains pieces of bran/vermiculite covered with a casing layer of peat/gypsum, while the "Macagnan medium" [16] contains dry broom material. When plugs of dikaryotic mycelia are transferred from agar culture to either of these two solid media and incubated at 25°C in Petri dishes, a network of hyphae initiates growth within and on the surface of the solid particles. Once the medium is well-colonized (similar to spawn-running in mushroom cultivation), basidiomata production is induced by opening the dishes, suspending the block of substrate (Figure 1D), and subjecting it to a regime of intermittent watering and a daily photoperiod of 10–12 h light.

When cultured in the "Griffith medium", mycelial mats of *M. perniciosa *isolate CP03 (see Methods) turned light-yellow four days after exposure to air and water, changing to reddish-pink after a further ten days, finally becoming dark-reddish pink until the onset of basidiomata development, some two to eight weeks later. These color changes were not uniform among parts of mycelial mats, varying according to irrigation intensity. The whitish aerial mycelium remained visible until the end of cultivation on some parts of the mycelial mats. Color changes also occurred in long-term stored mycelia at 25°C, however, basidiomata formation was never observed. Since mycelium color change was a pre-requisite for primordium formation, we standardized the collections according to their color.

**Figure 2:**
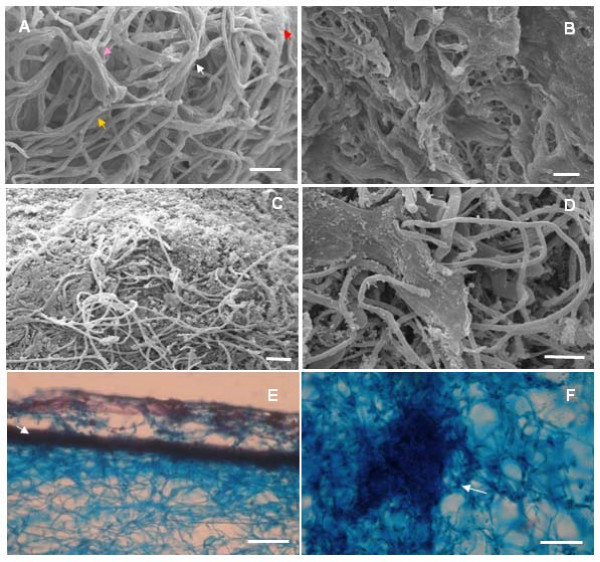
**Aspects of hyphal organization before fruiting of *M. perniciosa***. A-D: Scanning electron micrograph shows aerial hyphae. E-F. Section of mycelial mat of the "dark reddish pink" stage on dead cocoa branch, stained with Lugol and Safranine. A: Hyphae of mycelial mat in the white phase (Griffith medium). Note branched hyphae (yellow arrow), hyphal fusion (white arrow), thin layer apparently composed by cell wall materials (red arrow) and hyphae with irregular aspect (pink arrow; bar = 10 μm). B. Details of external hyphae after some days of exposure of mycelial mat to frequent irrigation. Note impregnated material in superficial hyphae (bar = 10 μm). C. Dark reddish pink mycelia with protuberance on the hyphae surface were they over layer the impregnated material, fanning out in ring shape (bar = 20 μm). D. Amorphous material recovering hyphae in differentiated primordium (bar = 10 μm). E. An outer layer (arrow) and aggregate aerial hyphae can be seen on the surface (bar = 0.12 mm). F. Hyphal nodule observed in reddish-pink mycelium (bar = 0.04 mm).

In an examination of the mycelial mats during the 32-day incubation period in Petri dishes, prior to incubation in the wetting/drying chambers, branched and agglomerated hyphae (mycelial cords) were observed fanning out on the surface of the substrate, appearing as long strands (Figure 2A, yellow arrow), with probable hyphal fusion along part of their length (Figure 2A, white arrow). At some points, hyphae were covered in a thin amorphous layer, apparently composed of plant cell wall material (Figure 2A, red arrow), as well as irregularly swollen and ornamented cells (Figure 2A, pink arrow). After exposure to water and air in the wetting/drying chamber, there appeared to be further agglomeration of hyphae into thicker structures, often covered with a layer of amorphous material (Figure 2B) and some raised areas with curved hyphae were also observed (Figure 2C). These changes were concurrent with the formation of yellow, reddish pink and dark-reddish pink pigmentation on the mat surfaces. In contrast, the mycelium on dry brooms already formed a dense layer at the white stage, probably due to the fact that this layer is formed in response to regular irrigation to which the brooms were subjected from the beginning of the experiment (Figure 1A and 1C).

Curved hyphae, leading to a possible hyphal fusion, were observed at this moment and in all distinct stages of the superficial mycelium, a pattern also observed in *Laccaria *spp [18]. Side-by-side hyphal branches evolved to larger plate-like structures in reddish pink mycelium (Figure 2B) and in mycelium forming the primordia apex (Figure 2D). These plate structures were not always continuous and some mycelial strands appeared empty or dry (not shown). A microscopic tissue section of reddish-pink mycelium in air contact revealed a distinctive mycelium layer with a mean thickness of 60 μm (Figure 2E, arrow), as well as internal net patterns of hyphae.

Similar patterns of hyphal growth were reported by Heckman et al. [28] in *A. bisporus *before basidiomata formation [28]. These authors recognized four morphological stages of mycelium and observed side-by-side hyphal fusions and the formation of hyphal wall ornamentation, which occurred in the first mycelial growth phase [28]. In the second stage, hyphal fusion led to the formation of structures called strands. Microscopic primordia were formed in the third stage in more compact masses, in areas of dense mycelial growth. At the fourth stage, primordia were visible to the unaided eye. Fused and ornamented hyphae as well as strands appeared in *M. perniciosa *before primordium development. Therefore, the process of primordium development of *M. perniciosa *was similar to that observed for *A. bisporus*, exept for the formation of an impermeable surface layer in hyphae and the type of hyphal ornamentation only observable in *M. perniciosa*.

The chemical composition of the impermeable surface layer was investigated. No reduced sugars, lipids and phenols were detected (data not shown). If these layers consisted of empty fused hyphae, chitinases were possibly active in this event. Lopes [29] observed an increased expression of chitinases in *M. perniciosa *in the reddish pink mycelium prior to basidiomata formation. It may also be possible that these areas are rich in hydrophobins, a protein required in basidiomata formation in several other fungi that form a thin outer layer on hyphae exposed to the air [30]. These proteins form an amphipathic layer between hydrophilic-hydrophobic interfaces, which protects the hyphae-inducing aerial mycelia [31]. An increased expression of hydrophobin-encoding genes was observed during mycelial mat growth of *M. perniciosa *[32].

Changes in pigmentation of the superficial mycelium of *M. perniciosa *were described by Purdy *et al*. [13] and by Griffith and Hedger [7]. In our experiments, changes in pigmentation were observed in mycelial mats washed in chambers until basidiomata emergence, indicating a correlation with basidiomata formation. The same color of the surface mycelium persists in the primordia, especially in the apices. The appearance of hyphal nodules coincided with the change in pigmentation from yellow to pink of the surface mycelium as described before (Figure 2F), and the primordia emerged after this color had darkened. Stronger pigmentation was observed on the primordia apex exactly at points of densely aggregated hyphae, which leads us to believe that pigmentation is correlated with hyphal aggregation. The term "hyphal nodules" has been used to describe the initial phases of basidiomata development [19] as well as for the nodules in the regions of the "initials" and in the morphogenesis-directing primordia [33].

**Figure 3:**
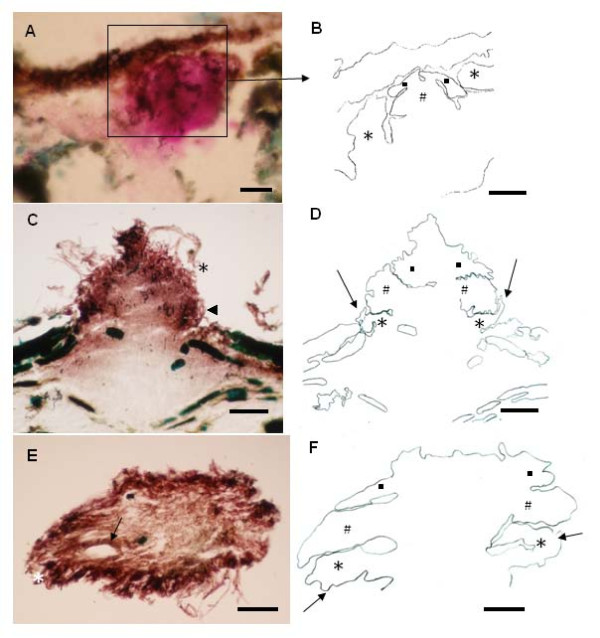
**Early developmental stages of *M. perniciosa *basidiomata**. **A**. Globose hyphal aggregate (initial primordium) under a superficial layer of mycelial mat (bar = 0.25 mm). **B**. Schematic drawing of the area marked in A showing the grouping of protective hyphae (*) laterally involving another more compact group (#). At the top another group of converging hyphae grows downwards (black squares) (bar = 0.12 mm). **C**. Tissue section showing an emerging undifferentiated "initial" (bar = 0.25 mm). **D**. Schematic drawing of C showing the expansion of marked hyphae presented in Figure 2B. The arrows indicate the same previous protective layer but the compact bulb has already overlapped it (bar = 0.25 mm). **E**. Another "initial" in a more advanced developmental state (bar = 0.25 mm). **F**. Schematic drawing of E showing protective hyphae placed in parallel positions and the laterally expanding bulb hyphae (arrows) (bar = 0.25 mm).

**Figure 4:**
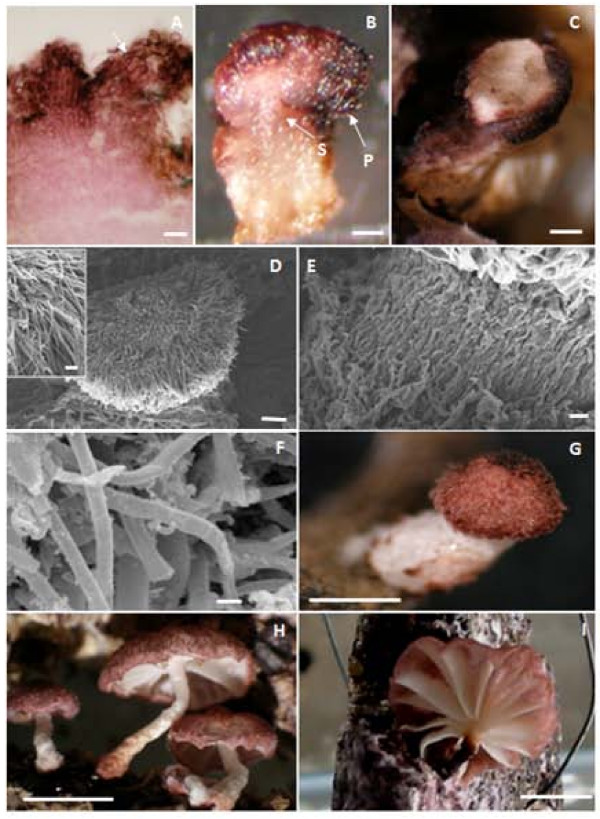
**Aspect of primordia of *M. perniciosa***. A. Section of initial primordium stained with Pianeze III. Note the globose form, the distance between the septa and the pigment impregnated within the hyphal cell wall (arrow; bar = 0.1 mm). B. Hand-cut section of a primordium showing the separation between the tissue that will develop the pileus (P) and the rod that will develop the stipe (S) (bar = 0.25 mm). C. Lamella appeared by digestion in areas of pileus (bar = 0.25 mm). D-F. Scanning electron micrograph. D. Differentiated primordium with radial growing hyphae in pileus (bar = 100 μm, on detail bar = 30 μm). E. Densely packed stipe hyphae (bar = 20 μm). F. Clamped hyphae of primordium (bar = 2 μm). G. Primordia extension stage (bar = 1 mm). H. Different primordia in extension stage (bar = 0.5 cm). I. Basidiomata obtained *in vitro *with exposed lamellae (bar = 1 cm).

Primordia of *M. perniciosa *appeared when the dense mycelial mat showed reddish-pink pigmentation. The first signal of primordial development was probably the appearance of primary hyphal nodules as well as internal local aggregations on dark pink-reddish mycelium (Figure 2F). Thereafter, hyphal interaction led to the formation of compact aggregates that can be considered an undifferentiated stage called initial primordium or secondary hyphal nodule [19] (Figure 3A). Hyphae belonging to such aggregates were short, large and strongly stainable with fuchsin acid, a substance present in Pianeze III solution, used to distinguish fungal from plant tissues (Figure 3A). The primordium emerged from within the surface mycelial layer (Figure 1E) as a well-defined protuberance (Figure 1F) with hyphae similar to those found in the aggregates (Figure 4A). The primordium initial (Figure 1F and Figure 3C) then underwent differentiation to form stipe, pileus (Figure 4B) and lamellae (Figure 4C). Hyphae of the primordium apex were cylindrical, with round apices and parallel growth, bending at the end distal to the pileus (Figure 4D, detail). Stipe hyphae were more compact, flat, growing vertically (Figure 4E). Amorphous material and clamped hyphae were also present on the apical primordium surface (Figure 2D and Figure 4F, respectively).

The various developmental stages of *M. perniciosa *basidiomata formation were very similar to those previously described in detail for *Agaricus sp. *[17], *C. cinerea *[19], *Mycena stylobates *[34] and *Laccaria *spp. [18]. Differentiation in *Agaricus *occurred at the initial stage to produce a bipolar fruiting body primordium [17,19]. This process appears to be conserved among *Agaricales *with slight differences between species. It was rather difficult to microscopically observe the hyphal nodule of the mycelial mats grown on "Griffith medium" due to the density of the hyphal layer. However, the primary hyphal nodule stages of *M. perniciosa *basidiomata were inferred from the presence of areas of intense localized ramifying hyphal aggregates in small nodules (Figure 2F). These nodules progressed to a globose aggregate, surrounded by a dense layer of amorphous material, an irregular arrangement of interwoven hyphae on the internal tissue of dry brooms stained green (Figure 3A), which can be considered the initial stage of hyphal aggregation. This hyphal agglomerate is distinguished by acid fuchsin which stains only living tissues [35]. Aggregates found in dark reddish-pink mycelium (Figure 2F) indicated a competent mycelium from which primordia may originate, similar to the aggregates in *Laccaria *sp., which would give rise to basidiomata [18].

Globose aggregates appeared on the surface with a protective layer covering a hyphal bulb (Figure 1E, *). Walther *et al*. [34] described a similar phenomenon in the initial development of *M. stylobates*. The initial formation of this layer can be observed in *M. perniciosa *(Figure 3A, arrow) that later covered the surface of the protuberant area (Figure 1E, *). Then, an initial emerged (Figure 1F and Figure 3C) and differentiated into a primordium, here referred to as the third stage (Figure 3E). It is likely that enzymatic digestion by chitinases [36] occurred in the hyphae of the outer layer, thereby allowing the "initial" to emerge as a dense layer, with amorphous material in the center of the protuberance. Differentiation continued leading to the formation of the lamellae (Figure 3E, arrow and Figure 4C) and later the pileus (Figure 4B). The apical region of initials formed the pileus and the basal region formed the stipe (Figure 4B). At the end of this stage the immature pileus and stipe (Figure 4G) could be seen with lamellae already established (not shown). Lamellae expanded after two to three days (Figure 4H), depending on sufficiently high moisture levels, as already observed for other basidiomycetes [17]. The hymenium was enclosed by incurved margins of the pileus, only being exposed when the basidiomata maturated (Figure 4G and 4H). Finally the stipe elongated and the pileus expanded to expose the hymenium for basidiospore liberation (Figure 4I). Basidiomata maturation was regulated by humidity and not all initial primordia progressed to form basidiomata (not shown).

Primordia emerged from 75 d after the exposure of substrate-grown mycelia to water and light in the humid chamber (Figure 1G). The first basidiomata were observed about 10 d after the first primordium was visible, but undifferentiated primordia were still present on the mat surface when basidiomata appeared. Density of primordia was high, their size not uniform and their production discontinuous, suggesting a programmed induction, as in plant inflorescences. The morphogenesis observed in the initials (Figure 3) resembled that of other Basidiomycota. Hyphae aggregated towards the surface and assumed a vertical position concurrent with an increase in diameter and compartment length (distance between septa) (Figure 3A and Figure 4A, arrow). These hyphae differentiated to form an agglomerate (Figure 3A) where they converged in an apical group (Figure 3B, #) and two lateral groups, growing in towards the bottom (Figure 3B, black square). A parallel bundle of hyphae with an inclination in direction to the center of the agglomerate was also observed (Figure 3B, *). This bundle diminished in length when the central aggregates increased in size; later, a lateral appendix to the primordium was observed (Figure 3D, arrows and *). Lateral groups (Figure 3D, #) increased in prominence during development, and the convergent hyphae at the agglomerate apex became vertically prominent (Figure 3D, black squares).

The lateral groups tended to bend downwards away from the apex (Figure 3C, *). A group of basal hyphae, however, bent upwards, supporting the hyphal extremity that bent downwards (Figure 3C, arrow and 3D, arrow). As the lateral hyphae expanded, the overlapping of these hyphae diminished (Figure 3E, * and 3F, arrows), increasing the space between these hyphal groups (Figure 3E, arrow). A micrograph of an emerged primordium (Figure 4C) shows a difference in opacity between hyphae, suggesting that a partial digestion led to the spaces between the lamellae. Another freehand section shows the lateral bending of hyphae and the differentiation of the stipe (Figure 4B). This primordium already possessed a differentiated hymenium (not shown).

Studies in *Agaricus *sp. and other edible fungi revealed a hemi-angiocarpous standard developmental stage [17,19], with a veil covering the primordium. In these fungi, a cluster of parallel and oriented hyphae emerges and forms the stipe and the pileus develops from the apical region. *Laccaria *sp. has a plectenchymal tissue from which the stipe originates, whilst the pileus arises from an apical prosenchymal tissue, as in *Agaricus *[18]. Similar structures were observed in *M. perniciosa *(Figure 3B). However, the development was pseudo-angiocarpous since the hymenium was protected by the immature pileus, and no inner veil was present (Figure 4B) [37]. The morphogenetic mechanism was classified as concentrated, based on the description of Reijnders [38] since defined globose primordia with a complex anatomy (Figure 3A) were formed. This is compatible with pileostiptocarpic development because stipe and pileus-originated elements were already present in the primordia at an early stage (Figure 4B).

### Genes related to the early development of *M. perniciosa *basidiomata

The molecular basis of cell differentiation that precedes basidiomata formation was recently investigated [17,19,39]. Developmentally regulated genes have been identified for some basidiomycetes such as *A. bisporus *[40], *C. cinerea *[19], *Pleurotus ostreatus *[41], among others. Moreover, the rapid increase of fully or partially sequenced genomes and ESTs from fungi already available in databanks allow the *in silico *identification of genes possibly involved in these processes [42,43]. However, the understanding of the direct association between these identified genes and their function in the initial development of basidiomata is still incipient. For example, the study of the ESTs of *P. ostreatus *led to the identification of pleurotolysins expressed specifically in the primordial stage. The function of these proteins is being studied, but their role in primordia formation is not yet elucidated [44].

Since the studies in *M. perniciosa *are also in an early stage, the identification of genes related to basidiomata development was a first step to establish a possible correlation between the developmental stages and their expression. The description of morphological changes in mycelium prior to the development of reproductive structures is a key step for subsequent morphogenetic studies and, at this point, helped in the search for genes related to these processes. So far, our contribution has been the analysis of the abundance of transcripts for some selected genes in specific moments during induction of fungal fruiting. Two independent but related tests were carried out. Using 192 genes from a library derived from mycelium in the fructification stage, a reverse Northern analysis, also known as macro array was performed, contrasting the early culturing with the final stage, when the first basidiomata appear. Additionally, a RT-qPCR was performed for 12 genes, analyzing their expression in each of the stages described in the above-described morphological studies.

The development of basidiomycetes such as *C. cinerea*, one of the best-studied to date [19], served as guideline underlying the choice of the genes. In the case of these fungi, fructification seems to occur in genetically pre-conditioned mycelium and in response to nutrient deficiency, as well as to stimuli such as alternating light/dark, humidity and CO_2 _concentration [19]. Based on these studies, genes were selected and identified in the available library.

### Expression profiles of genes involved in basidiomata development by macroarray

**Figure 5:**
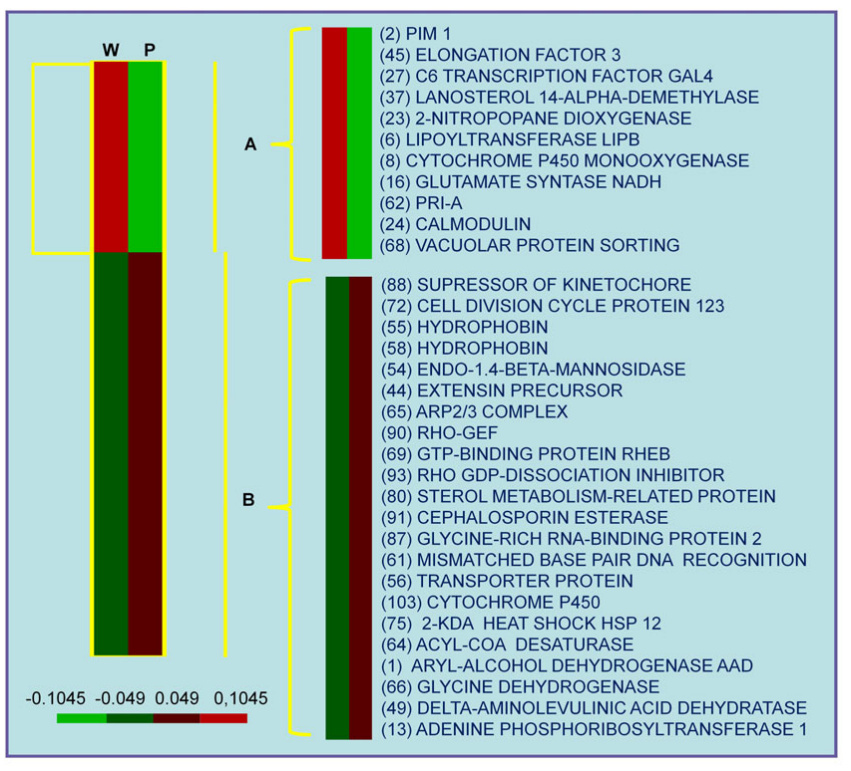
**Genes expressed differentially in white mycelia and mycelia with primordia A**. Hierarchical clustering illustrating groups of 192 *M. perniciosa *genes coordinately expressed at the moment of fruiting versus white mycelium stage by macrorray assay. The column W represents samples of white mycelium stages and P the primordium stage. For each gene, the medium mRNA levels represented by red or green, indicating up-regulation or down-regulation, respectively. The legend indicates the corresponding values of intensity. Two groups are formed: A = higher gene expression in 'white' mycelium and B = higher expression in mycelium with 'primordia'. On the right are examples of genes evaluated in each group.

A macroarray analysis was performed with 192 genes encoding putative proteins involved in fruiting, to detect differences in their expression profile between mycelia in white and primordial phases, which would allow their identification as induced or repressed at these two contrasting developmental stages (Figure 5). ESTs were obtained from a full-length cDNA library, previously constructed from mycelia, primordia and mature basidiomata collected during fructification (Pires et al., unpublished data) and selected based on their similarity with known conserved genes. The complete list of the selected genes is shown in Table S1 [see Additional file 1] as well as the fold change values obtained by comparing the results of each spot in the 'white' and ' primordia ' stages. A classification based on the likely functions of these gene products was performed as described by Gesteira *et al. *[45], to deepen the understanding of the participation of these genes in the fructification process of *M. perniciosa*. The Table S1 [see Additional file 1] shows also some genes for which the increase of transcripts in the primordial stage compared to the white phase was significant by the Student's t test of means.

The macroarray analyses give us an overview of gene activity during fruiting in *M. perniciosa*. We discriminated 192 genes in two expression patterns: group I, containing up-regulated genes in the white mycelium phase and group II, containing up-regulated genes in the primordia mycelium phase (Figure 5). Some genes are noteworthy because previous descriptions report their participation in the fruiting process of other fungi. In this trial, hydrophobins were represented by four clones and three of them showed increased expression during the primordial stage.

Hydrophobins are cysteine-rich proteins specific for filamentous fungi, capable of generating amphipathic films on the surface of an object [31]. They are related to a broad range of growth and development processes, among them the formation of aerial structures [46]. At least five *M. perniciosa *hydrophobin-encoding genes have been identified [27]. The differences in expression in mycelial mat cultures for basidiomata production were considerable. Unlike four other genes for hydrophobin, one gene was shown to have increased expression in the presence of primordia [32] and two were identified in a compatible *M. perniciosa-T. cacao *cDNA library derived from green brooms [45].

Studies in other fungi show that hemolysin expression is specifically increased in the presence of primordia [47], but in this experiment there was no significant increase in the expression of the genes that encode for aegerolysins. Only one gene for pleurotolysin A decreased significantly. On the other hand, genes encoding cytochrome P450 mono-oxygenase and a heat shock protein had increased expression in the primordial stage, which may indicate the induction of fruiting in response to stress [17]. Cytochrome P450 mono-oxygenases ('P450s') are a super-family of haem-thiolate proteins that are involved in the metabolism of a wide variety of endogenous and xenobiotic compounds [48]. In *C. cinerea*, the cytochrome P450 similar to CYP64 is most expressed in pilei and seems to be involved in the synthesis route of aflatoxins that seem to be important for fruiting in *Aspergillus *spp. [17].

The appearance of primordia coincided with the decrease of transcripts for calmodulin and increased expression for genes coding for signaling proteins such as RHO1 guanine nucleotide exchange factor (RHO-GEF), RHO GDP-dissociation inhibitor, GTP-binding protein RHEB homolog precursor, indicating that signaling is most likely mediated by fruiting-associated proteins of the Ras family. Additionally, the genes for cellular transport of glucose and gluconate were clearly more significantly transcribed at the primordial stage [see additional file 1], while a probable transcription factor GAL4 decreased. This indicates that glucose depletion of the medium, which occurs throughout the culture, must be important for fructification and must be related to cAMP signaling [49]. Gene *gti1*, encoding an inducer of gluconate transport in *Pseudomonas aeruginosa*, controls glucose catabolism, increasing the low-affinity transport system of glucose [50]. The glucose transporter present in this test is rather similar to the high-affinity glucose transporter SNF3, although this has not been confirmed experimentally [51]. Glucose metabolism can be related to fructification [17].

The increase of gene transcripts for vacuolar ATP synthase, phospholipid-transporting ATPase and reductase levodione also indicates that nutrient uptake during the primordial stage serves to form nutrient reserves prior to basidiomata elongation [17]. This is confirmed by the increase of transcripts for several genes of primary and secondary metabolism that may be related to the synthesis of glycerol and lipids. In *C. cinerea *reserves are remobilized and glycogen accumulated in the primordial stage [19].

The expression of three genes related to cell division was significantly higher, two for a 123 kD protein of cell division (Cdc123) and one encoding a suppressor of kinetochore, and one *PIM1 *gene was significantly less expressed in the primordial stage. Cdc123 proteins are regulators of eIF2 in *Saccharomyces cerevisiae *and are regulated by nutrient availability [52]. This simultaneous increase indicates the predominance of phase G1 of cell division. As the formation of spores occurs in already differentiated primordia, it is likely that the collected phase contains a larger number of non-differentiated primordia.

There was also a significant increase of six genes of unknown function, one of them showing no similarity with any sequence in the available public data banks.

### Expression analyses of genes involved in basidiomata development by RT-qPCR

**Figure 6:**
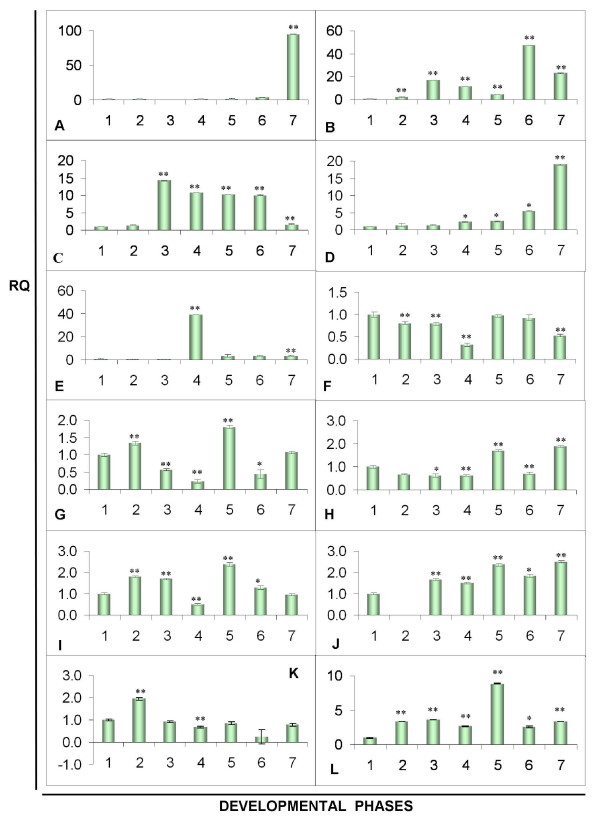
**RT-qPCR of genes expressed in different phases during the culture of *M. perniciosa *in basidiomata-inducing medium**. A – Mp*PRIA1*; B – Mp*PRIA2*; C – Mp*PLYB*; D – Mp*RHEB*; E – Mp*GLU*; F – Mp*ADE*; G – Mp*CPR*; H – Mp*RHO1-GEF*, I – Mp*MBF*; J – Mp*RAB*; K – Mp*CYP*; L – Mp*RPL18*. In Y axis values of RQ using primers constructed for each gene and in axis X corresponding samples of RNA originated from mycelia in the following stages: 1 = cDNA of mycelium white stage, 2 = cDNA of yellow mycelium stage, 3 = cDNA of reddish pink mycelium stage, 4 = cDNA of reddish pink mycelium before stress, 5 = cDNA of reddish pink mycelium after stress, 6 = cDNA of mycelium with primordia and 7 = cDNA of basidiomata. RQ = relative quantification measured by ddCt. (*) – significant at 5% probability, (**) – significant at 1% probability by the statistical t test.

The gene expression profile obtained by the macroarray in two distinct phases suggested physiological changes in mycelia prior to basidiomata production. However, more detailed analyses should be performed to monitor the expression of key genes (previously described in the literature as involved in basidiomata development). Quantitative PCR is an accurate technique to analyze gene expression. It is 10,000 to 100,000 times more sensitive than RNase protection assays and 1,000 times more sensitive than dot blot hybridization [53]. Therefore, a more detailed RT-qPCR analysis was performed with 12 ESTs in order to observe a possible relationship between transcript levels of all samples collected (Figure 6). RNA was obtained from mycelium samples of all seven developmental stages: white, yellow and reddish pink phases, before and after stress, and during basidiomata formation.

The hypothesis that nutrient depletion might act as a signal for fructification was confirmed since some genes related to primary metabolism and to nutrient uptake were down-regulated when primordia emerged. Conversely, gene expression related to nutrient recycling and stress response increased during this phase, as did the expression of genes directly involved in cell development (Figure 3). The relative expression of the 12 genes in stages that precede fructification helped elucidate the correlation between nutrient depletion and fructification (Figure 6) since the genes Mp*RHEB*, Mp*RHO1-GEF*, Mp*ADE*, Mp*MBF*, and Mp*RAB *putatively involved in signaling are associated with internal perception of the signals triggered by nutrient depletion and other stresses, which was noticeable before the primordia appeared. The putative gene Mp*RHEB *is associated with growth regulation probably during nitrogen depletion [54]. Its expression in *M. perniciosa *increased in reddish pink mycelium, immediately before stress and continued at a high level until the beginning of the primordial and basidiomata phases (Figure 6D). The expression of the high-affinity transporter Mp*GLU *[51] peaked in this mycelium before stress (Figure 6E), strongly indicating a nutritional deficit, namely low external glucose concentration. Moreover, expression of Mp*CPR *and Mp*CYP *was low during this period (Figure 6G and 6K), indicating a lower basal metabolism [48]. The expression of Mp*RAB *(Figure 6J) may indicate nutrient remobilization, since it is involved in intracellular traffic [55,56].

During the water stress applied to trigger *in vitro *fructification expression of some genes peaked. Transcripts of putative Mp*MBF *(multi-protein-bridging factor), a co-activator related to tolerance to abiotic stresses in plants [57], increased 2.4-fold (Figure 6I). Other genes with increased expression during this stress period were Mp*RHO1-GEF *(Figure 6H), involved in signaling for the regulation of polarized growth [58] and Mp*RPL18 *(Figure 6L) involved in protein synthesis. Involvement of signalization, probably cAMP-mediated, is likely due the expression of adenylate cyclase that decreased in the yellow and reddish-pink mycelial phases, to return to the original levels observed on white mycelium just after the stress period (Figure 6F). As adenylate cyclase is subject to post-translational regulation, studies of enzymatic activity would be necessary to confirm this hypothesis. The gene *p-rho/gef *is, therefore, possibly correlated with cAMP pathways. Repression of the glucose transporter coincided with the repression of the adenylate cyclase gene, which also indicates cAMP signaling. In *S. pombe *the glucose levels are regulated by adenylate cyclase [59] and in *Sclerotinia sclerotiorus *the development of reproductive structures is negatively regulated by cAMP [60]

### Putative aegerolysins and pleurotolysin B of *M. perniciosa *are differentially expressed during fructification

As described for other fungi, probable hemolysins are highly expressed at the fructification stages [47,61]. We identified three putative genes involved in fructification, two more closely related to the identified *AA-Pri1 *or *PriA*s of *Agrocybe aerogerita *and *P. ostreatus*, respectively, and one more closely related to pleurotolysin B, also identified in *P. ostreatus*. Their different expression profiles suggested that they are different genes (Figure 6A to 6C). The expression of Mp*PRIA1*encoding a putative aegerolysin, decreased in the yellow- and reddish pink-mycelium phases, and also before stress, but increased 4.3-fold in mycelia with primordia, and about 90-fold in the basidiomata, compared to the white mycelium stage (Figure 6A). The expression of the putative hemolysin-encoding gene Mp*PRIA1 *increased 17-fold at the reddish pink mycelium stage, but decreased 11-fold before stress, 4-fold in stressed mycelia, and 47.4-fold in mycelia with primordia. The transcripts of Mp*PRIA2 *increased 23-fold in basidiomata, but were lower in mycelia with primordia (Figure 6B). The transcripts of gene Mp*PLYB*, corresponding to a pleurotolysin B, increased 1.4-fold in the yellow mycelium stage, 15.2-fold in reddish pink mycelia, and remained at high levels in the mycelia before stress (11.7-fold), when stressed (11.2-fold) and in mycelia with primordia (10.1-fold), but decreased in basidiomata, where it was only 1.6 times higher than in white mycelia (Figure 6C).

Hemolysins, already identified in some bacteria and fungi, comprise a cytolytic protein family, whose members appear abundantly during primordia and basidiomata formation [47,58,61,62]. Mp*PRIA1 *and Mp*PRIA2 *have homologous regions but seem to correspond to two individual genes whose expression coincides with the morphological differentiation of primary hyphal nodules from primordia. These hemolysins may contribute to the process of hyphal aggregation [61] as their expression occurred, although at low levels, before the appearance of primordia, when hyphae became globose for the formation of the "initials". This stage coincides with the reddish pink mycelium stage, where hyphal nodules are detectable. The exact function of these proteins remains unclear, but their involvement in programmed cell death (PCD), as proposed by Kues and Liu [17], seems rather unlikely because ostreolysins have lytic function, acting in cholesterol- and sphingomyelin-containing membranes [63] at a pH between 7 and 8 [64], which is not usually found in fungal cells.

The known fungal hemolysins have some variations in amino acid sequences, but all share the conserved domain aegerolysin (code PF06355 by Pfam database [65]). Aegerolysin *Aa-Pri1 *from *A. aegerita *has the same molecular weight as the 16 kDa ostreolysin of *P. ostreatus *and is mainly expressed in the initial stage of primordium formation. PriA (or pleurotolysin or PlyA) of *P. ostreatus *forms a subfamily with the aegerolysin superfamily, which includes the Asp-hemolysins of *Aspergillus fumigatus*, and some hypothetical proteins of *Clostridium bifermentans*, *P. aeruginosa *and *Neurospora crassa*. *P. ostreatus *hemolysin consists of multiple components with isoforms A and B that assemble to a protein complex that leads to the formation of transmembrane pores (diameter 4 nm), specifically allowing lysis of cholesterol and sphingomyelin-containing membranes [63]. Isoform A, called PlyA [17 kDa *PlyA*] has 138 amino acid residues whereas the 59 kDa isoform B polypeptide (PlyB) consists of 538 amino acids.

**Figure 7:**
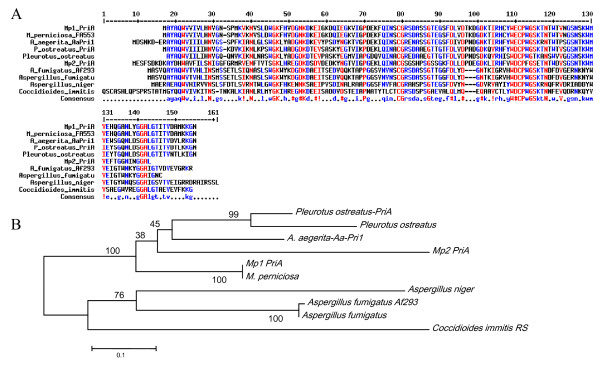
**Comparison between *M. perniciosa *aegerolysins and other fungi**. **A **– Alignment for similarity between ORFs of the two probable aegerolysins of *M. perniciosa *(Mp*PRIA1 *and Mp*PRIA*2) and aegerolysins of *M. perniciosa *FA553 (gbEEB90416.1), *A. aegerita *(spO42717.1), *P. ostreatus *(PriA – gbAAL57035.1 and ostreolysin – gbAAX21097.1), *A. fumigatus *Af293 (XP 748379.1), *A. fumigatus *(gbBAA03951.1) *C. immitis *RS (XP_001242288.1) *A. niger *(XP_001389418.1). Strictly conserved residues are shown in black and similar residues in gray. Consensus symbols: ! is any of IV, $ is any of LM, % is any of FY, # is any of NDQEBZ. Domain PF06355 (aegerolysin family) is present in Mp*PRIA1 *(residues 4–136, score 8.7e-61) and Mp*PRIA*2 (residues 29–135, score 4.2e-34). **B**. Phylogenetic analysis of the probable aegerolysin genes of *M. perniciosa *with above-cited sequences. Evolutionary history was inferred using the Neighbor-Joining method. The bootstrap consensus tree inferred from 1000 replicates is taken to represent the evolutionary history of the analyzed taxa.

**Figure 8:**
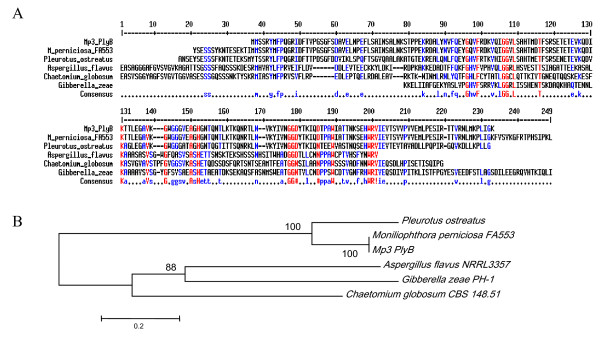
**Comparison between *M. perniciosa *pleurotolysin and other fungi**. **A **– Alignment for similarity between ORFS of the one probable pleurotolysin B of *M. perniciosa *(Mp*PLYB*) and hypothetical proteins of *M. perniciosa *FA553 (*gb *EEB89936.1), *P. ostreatus *(*gb *BAD66667.1), *G. zeae *PH-1 (XP_390875.1), *A. flavus *NRRL3357 (gbEED49642.1), *C. globosum *CBS 148.51 (XP_001227240.1). Strictly conserved residues are shown in black and similar residues in gray. Consensus symbols are used similarly as in Figure 7. Domain MAC/Perforin (PF01823) is present in Mp*PLYB *(residues 1 to 258, score -35,2). **B**. Phylogenetic analysis of the probable pleurotolysin B gene of *M. perniciosa *with above-cited sequences. The evolutionary history was inferred using the Neighbor-Joining method. The bootstrap consensus tree inferred from 1000 replicates is taken to represent the evolutionary history of the taxa analyzed.

The two aegerolysin ESTs expressed by *M. perniciosa *constitute two distinct genes (Figures 7 and 8). Mp*PRIA1 *has an ORF of 417 bp with an intron at position 103 whereas the ORF of Mp*PRIA2 *is 406 bp long with an intron at position 134 (data not shown). Both have a conserved aegerolysin domain between residues 4 to 136 (Mp*PRIA1*) and 29 to 135 (Mp*PRIA2*) and can be aligned with a hypothetical protein MPER_11381 (gbEEB90416.1) (Figure 7A) and MPER_04618 (gbEEB96271.1 – not shown) of *M. perniciosa *FA553 and proteins described as aegerolysins of *A. aegerita *(spO42717.1), *P. ostreatus *(PlyA – gbAAL57035.1 and ostreolysin – gbAAX21097.1), *A. fumigatus *Af293 (XP 748379.1), *A. fumigatus *(gbBAA03951.1) *Coccidioides immitis *RS (XP_001242288.1) *A. niger *(XP_001389418.1) (Figure 7A). The evolutionary distance between these putative aegerolysins and above-cited aegerolysin of the Gene Bank database was estimated (Figure 7B). The distances were shorter between Mp*PRIA1 *and Mp*PRIA2 *and aegerolysins of *Pleurotus *and *Agrocybe *than between MpPRIAs and Asp-hemolysins and ostreolysins of *Aspergillus*.

The Mp*PLYB *ORF has 576 bp and two introns (not shown) at positions 211 and 408 corresponding to the genomic DNA of *M. perniciosa *in position 178 to 368 of the sequence deposited in GeneBank (accession no. ABRE01016965). The Mp*PLYB *ORF is more similar to hypothetical proteins of *M. perniciosa *FA553 (gb EEB89936.1) and pleurotolysin B gene described for *P. ostreatus *(gbBAD66667.1) and it can be aligned with proteins described as *Gibberella zeae *PH-1 (XP_390875.1) *A. flavus *NRRL3357 (gbEED49642.1) and *Chaetomium globosum *CBS 148.51 (XP_001227240.1) (Figure 8A). A conserved transmembrane domain MAC/Perforin [PF 01823] occurs between residues 1 and 258. The evolutionary distance between these putative pleurotolysin B and above-cited proteins of the Gene Bank database was estimated (Figure 8B). The distance was shortest between MpPlyB and pleurotolysin B of *Pleurotus*, while the similarity with hypothetical protein MpER_11918 of *M. perniciosa *was highest.

## Conclusion

Our analysis of gene expression is an initial approach to correlate gene expression with distinct developmental stages of *M. perniciosa *basidiomata. Gene expression profiles in mycelia before basidiomata induction indicate that the observed morphological changes correlate with induction of genes known to be involved in the development of new macroscopic structures in other fungi. An involvement of a glucose depletion-dependent cell signaling is suggested by the regulation of adenylate cyclase and glucose transporter genes. However, other up-regulated genes may be responsible for the formation of hyphal nodules, redirecting cytoskeleton modeling, hyphal thickness or nutrient uptake, and most of them may be essential for the maintenance of basidiomata. Our data provide new information about the development of basidiomata in *M. perniciosa *and identify a set of genes probably involved in this process. This information may be useful for further studies towards a more complete understanding of the cell processes and genetic, physiological and environmental controls leading to basidiomata initiation. Once the key genes that determine growth and development of *M. perniciosa *are known, strategies can be provided for an enhanced control of this phytopathogen and for a successful monitoring of witches' broom disease in *T. cacao*.

## Methods

### Fungal strains and growth conditions

A considerable number of observations of the early primordia development were made in infected brooms collected from cocoa plantations in Itajuípe (14° 40' 43" S, 39° 22'31" W), Bahia, Brazil. The brooms were kept in a moist chamber and basidiomata formation was induced. Briefly, they were soaked for 1 h in 1% benomyl solution (Sigma Chemical Co., St. Louis, USA), to kill the ascomycete fungi present on the broom surfaces, hung in a chamber (12:12 h light:dark) and sprayed with de-ionized water for 1 min/h for each 24 h period. *M. perniciosa *strain CEPEC 1108 (designated CP03) of the C biotype of *M. perniciosa *was also used for morphological studies. Mycelial starter cultures from the culture collection of the Cocoa Research Center (CEPEC, Ilhéus, Bahia, Brazil) were grown on PDA (Potato Dextrose Agar) for three weeks in the dark, at room temperature. Basidiomata were obtained from mycelial mats, as described by Griffith and Hedger [7] with the modifications introduced by Niella *et al. *[15]. A solid bran-based medium was prepared (50 g wheat flour; 40 g vermiculite; 6 g CaSO_4 _× 2H_2_O, 3 g CaCO_3 _and 120 mL distilled water; moisture content 65–70%, pH 7.0–7.5). The mixture was placed in Petri dishes, covered with aluminum foil and autoclaved twice for 90 min (121°C). The cooled medium was inoculated with two 5-mm disc plugs from 1 to 3-week-old mycelium, grown on 2% PDA medium. Cultures were incubated at 25°C in the dark. After mycelia had completely colonized the surface of the bran medium (usually 3–4 weeks), cultures were covered with a 5-mm thick layer (5–10 g per culture), composed of 200 g coarse peat, 50 g CaCO_3_, 50 g vermiculite and 125 mL distilled water (moisture content 70–75%, pH 7.0–7.5). These cultures were incubated for 3 to 4 weeks at 25°C in the dark and then hung vertically in a broom chamber [14], and maintained at 23°C ± 2°C for 75 d. Irrigation consisted of spraying de-ionized water daily for 7 h with a 12 h period of fluorescent warm white light (65–80 W). After 30 d in the chambers, the irrigation was suspended for 7 d, a procedure routinely used to induce fructification.

### Microscopic analyses

The preparation of mycelial mat samples for light microscopy was conducted according to standard histological methods [66]. For histological studies of basidiomata development at various stages, samples were fixed after collection by dehydration in a gradient of ethanol/tertiary butyl alcohol series (50 to 100%) for 2 h each, and thermally embedded in paraffin (melting point 56.5°C; Paraplast plus; Fisher Sci. Co., Pittsburgh, USA). The embedded tissues were radially cut (5 to 14 μm thick) with a rotary microtome. Serial sections were thermally mounted on microscope slides coated with Haupt's adhesive and 4% formalin [67]. The sections were immersed/rinsed three times in 100% xylene and passed through a series of xylene and absolute ethyl alcohol (EtOH) 1:1, absolute ETOH, and 70% ETOH. Some sections were stained with Pianeze III-B stain [68,69]. This procedure specifically stained soluble and insoluble proteins red with acid fuchsin and non-living material, i.e. polysaccharides and phenol, green to dark green [35]. Other sections were stained for 1 h with 1% astra blue and then for 1 h with 1% safranin. Macroscopic and tissue analysis was performed in a stereomicroscope (Olympus magnifying glass model SZ2-LGB) and an optical microscope (Olympus model CX41RF), both connected to digital cameras (Olympus model NOC 7070). For histochemical tests, sections of mycelial mats were checked by Fehling' Test [70] to detect reduced sugars, by Sudan III solution [71] to detect lipids and by Floroglucinol Acid solution [66] to detect phenolic compounds.

For scanning electron microscopy (SEM), samples were fixed in FAA (5% formaldehyde; 5% acetic acid; 63% ethanol), and dehydrated in increasing acetone solutions (30 to 100%), for 15 min at each concentration. Sections were dried to the critical point, mounted in stubs, and covered with gold before SEM analysis (Model LEO 54× (Zeiss), at the State University of Feira de Santana (Feira de Santana, Bahia, Brazil).

### Fungal strains, sampling, growth conditions for molecular analysis and RNA isolation

*M. perniciosa *strain FA553 (Cp02), sequenced by the WBD Genome Project [27] was used for macroarray and RT-qPCR analyses. Growth conditions were described as above except for some details: the chamber was a glass box (40 × 30 × 30 cm) with hooks on the lid underside. Units of mycelial mats were suspended on these hooks and washed aseptically. Temperature and light were as mentioned above. Samples were collected in the different pigmentation phases: white, yellow, reddish-pink, reddish-pink before stress and reddish-pink mycelium after stress (10 d without irrigation); mycelium containing primordia, and basidiomata (Figure 1G). Individual samples of CP02 were processed using the RNAeasy Plant Midi Kit (Qiagen, Valencia, USA). The RNA samples were qualitatively and quantitatively analyzed by denaturing formaldehyde/agarose gel electrophoresis and optical density was determined [72]. Aliquots of each sample were stored at -80°C until analysis. Figure 1G summarizes sampling for RNA extractions.

### cDNA library construction and analysis of differential gene expression by macroarray

The macroarray membrane was spotted with 192 cDNA clones in duplicate, which were selected from a cDNA library based on their putative role in basidiomata development in other fungi and their involvement in nutrient depletion and cell signaling. For the cDNA library construction, the *M. perniciosa *strain CEPEC 1108 (CP03) was cultured as previously described and mycelium samples in white, yellow, reddish-pink, dark reddish pink and primordium stages, as well as from basidiomata were used to construct a full-length, non-normalized cDNA library. Total RNA was extracted from samples using RNAs in RNA Plant Midi Kit as described by the manufacturer (Qiagen) and after quantification, 1 μg was used to construct the library using DB SMART Creator cDNA library as described by the manufacturer (Clontech). cDNA strands longer than 400 bp were cloned directionally into the pDNR-LIB plasmid. ElectroMAX™ DH10BTM cells (Invitrogen) were transformed and colonies selected and grown in 96-well microtiter plates in LB, 40% glycerol medium containing 30 μg/L chloramphenicol and stored at -80°C. A total of 2,759 clones were sequenced using a capillary sequencer (Mega Bace 1000, GE Healthcare). After the filtering, trimming, and clustering processes the 1,533 obtained ESTs were evaluated based on functional annotation. The cDNA fragments used to spot the macroarray membrane were amplified by PCR using M13 primers [forward 5'-CAGGAAACAGCTATGAC-3' and reverse 5'-GTAAAACGACGGCCAG-3'] that annealed to the vector pDNR-LIB (Clontech), transferred in duplicate to membranes (Hybond N+, Amersham Biosciences) [72] and fixed using a UV crosslinker (Spectronics Corporation). For macroarray hybridization, two distinct RNA pools were used: one cDNA mixture of three distinct biological samples from the initial cultivation phases on artificial media (white phase), and another cDNA mixture of three distinct biological samples from the primordial stage. The membrane was hybridized twice with each cDNA pool. Labeling (400 ng of each cDNA pool), pre-hybridization (4 h), hybridization (2.5 h) and signal detection were performed as recommended by the manufacturer of the Alkaphos kit (GE Healthcare). The membranes were exposed to X-Omat (Kodak) film for 2.5 h and the images captured using the Scanner Power Look 1120 UDS (Amersham Biosciences) and analyzed with BZ Scan [73]. The presence or absence of the signal, as well as the intensity, was registered for each individual spot. Global normalization and clustering of the generated intensities, using software Cluster version 3.0 [74]. The default Cluster for normalization was performed eight times, with genes centralized by average. A total clustering of genes was made by the uncentered method (Pearson correlation). This value used in hierarquical clustering represents the average intensity of each gene. Student's t-test, was used after global standardization and before clustering to establish a comparison between means. The values significant at 5% probability and the genes accession numbers are shown in Table S1 [see Additional file 1] together with the fold change values based on the means generated after normalization by Cluster 3.0 software.

### Quantitative analyses of reversed transcripts (RT-qPCR)

During the growth period in artificial medium, 12 selected genes were analyzed based on their expression pattern derived from the macroarray. The following genes were selected from the EST data base http://www.lge.ibi.unicamp.br/vassoura encoding the proteins: three putative hemolysins (CP03-EB-001-020-G09-UE.F; CP03-EB-001-008-C10-UE.F; CP03-EB-001-024-G03-UE.F), a putative 60S ribosomal L18 protein (CP03-EB-001-001-E05-UE.F), a putative Rho1/GEF (CP03-EB-001-012-F03-UE.F), a putative Rab (Ras family) (CP03-EB-001-020-F11-UE.F), a putative multi-protein-bridging factor (CP03-EB-001-025-E06-UE.F), a putative Ras-GTP-binding protein Rhb1 (CP03-EB-001-005-E11-UE.F), a putative glucose transporter (CP03-EB-001-015-G10-UE.F), a putative cytochrome P450 (CP03-EB-001-025-D09-UE.F), a putative adenylate cyclase (CP03-EB-001-025-C05-UE.F), and a putative NADPH-cytochrome P450 reductase (CP03-EB-001-001-B10-UE.F). A putative polyubiquitin (CP03-EB-001-020-H08-UE.F) was used as reference gene. All PCR primers (MWG, Imprint Genetics Corp) were designed using the GeneScript online Real-Time Primer Design tool https://www.genscript.com/ssl-bin/app/primer [see Additional file 2]. One microgram of total RNA treated with RQ1 DNAse I (Invitrogen) was reverse-transcribed using Power Script (Invitrogen) at a final volume of 20 μL. The primer Tm was set at 59°C to 61°C and the amplicon sizes ranging from 100 to 105 bp. Quantitative PCR was performed using SYBRGreen^® ^(Invitrogen) for the detection of fluorescence during amplification, and assays were performed on an ABI PRISM 7500 Sequence Detection System (SDS) coupled to the ABI PRISM 7500 SDS software (Applied Biosystems, Foster City, USA), using standard settings. A 20 μL RT-PCR reaction consisted of 2 μL SYBRGreen 1× (Applied Biosciences), 1× PCR buffer, 200 mM dNTPs, 3 mM MgCl2, 1/2 50× Rox, 200 nM each primer and 10 μL single-stranded cDNA. The thermal cycling conditions were 50°C for 2 min, then 94°C for 10 min, followed by 40 cycles of 94°C for 45 s, 57°C for 35 s for annealing, and 72°C for 35 s. A dissociation analysis was conducted after all amplifications to investigate the formation of primer dimers and hairpins. Melting temperatures of the fragments were determined according to the manufacturer's protocol. No-template reactions were included as negative controls in every plate. Sequence Detection Software (Applied Biosystems, Foster City, USA) results were imported into Microsoft Excel for further analysis. Raw expression levels were calculated from the average of the triplicate ddCT (RQ) values using the standard curve obtained for each primer pair (ABI PRISM 7500 Sequence Detection System User Bulletin #2). A non-parametric *t *test was performed in order to compare the expression values obtained for each gene between the samples.

### Molecular analyses of aegerolysin genes

The two putative aegerolysin genes (Mp*PRIA1 *and Mp*PRIA2*) and one putative pleurotolysin B (Mp*PLYB*), were analyzed by aligning ESTs and genomic sequences using Clustal W (EBI) [75]. The contigs were screened for conserved domains and for introns using ORFINDER software (NCBI-http://www.ncbi.nlm.nih.gov/projects/gorf). The amino acid sequences generated from the most likely ORFs were aligned against four sequences available at the UNIPROT database [76] using Multalign [77]. The evolutionary history was inferred using the Neighbor-Joining method [78]. The evolutionary distances were calculated following the Poisson correction method [79] and expressed in units of number of amino acid substitutions per site. All positions containing gaps and missing data were eliminated from the dataset (complete deletion option). There were a total of 116 positions in the final dataset. Phylogenetic analyses were conducted in MEGA4 [80]. Branches corresponding to partitions reproduced in less than 50% bootstrap replicates were collapsed. The percentage of replicate trees in which the associated taxa clustered together in the bootstrap test (1000 replicates) is shown next to the branches [81].

## Authors' contributions

ABLP -Fungus culturing, RNA extraction, cDNA library construction, microscopy tissue preparations, macroarray and RT-qPCR analyses, electronic microscopy analyses and manuscript drafting. MMS – Fungus maintenance, RNA extraction and cDNA library construction. KPG – Fungus maintenance, microscopy tissue preparations and manuscript drafting. DCS – microscopy slide preparations and biochemical tests. RFP and JSMF – macroarray construction. CVD – macroarray construction and RT qPCR analyses. AGN – scanning microscopy analyses and manuscript draft preparation. MB – manuscript preparation and result interpretation. JCMC and GAGP – headed and promoted the Project, manuscript elaboration. All authors read and approved the final manuscript.

## Supplementary Material

Additional file 1 **Supplemental Table S1**. Differentially expressed genes between white and primordia stages evaluated by macro-arrays and Gene Bank accession numbers. Click here for file

Additional file 2 **Supplemental Table S2**. Oligonucleotides used in this study with corresponding gene function. Click here for file
